# Clinical effect of minimally invasive aspiration and drainage of intracranial hematoma in the treatment of cerebral hemorrhage

**DOI:** 10.12669/pjms.38.1.4618

**Published:** 2022

**Authors:** Chao Deng, Yuanling Ji, Wei Song, Jingfang Bi

**Affiliations:** 1 Chao Deng Department of Neurosurgery, Taian City Central Hospital, Shandong, 271000, China; 2 Yuanling Ji Department of ICU, The Second Affiliated Hospital of Shandong First Medical University, 271000, China; 3 Wei Song Department of Neurosurgery, Taian City Central Hospital, Shandong, 271000, China; 4 Jingfang Bi Department of Neurosurgery, Taian City Central Hospital, Shandong, 271000, China

**Keywords:** Cerebral hemorrhage, intracranial hematoma aspiration and drainage, Neurological function, prognosis

## Abstract

**Objectives:**

To explore the clinical value of minimally invasive aspiration and drainage of intracranial hematoma in the treatment of cerebral hemorrhage.

**Methods:**

Seventy-eight patients with cerebral hemorrhage who were treated in the Taian City Central Hospital and the Second Affiliated Hospital of Shandong First Medical University between June 2018 and December 2019 were selected. The patients were randomly numbered and divided into two groups by drawing lots, 39 in each group. The control group was treated with the traditional internal medicine conservative therapy, and the observation group was treated with minimally invasive intracranial hematoma aspiration and drainage. The indexes of the two groups were compared.

**Results:**

The efficacy rate of the observation group was significantly higher than that of the control group, and the difference was statistically significant (P<0.05). The National Institutes of Health Stroke Scale (NIHSS) score of the observation group was lower than that of the control group after treatment, and the difference was statistically significant (P<0.05). After treatment, the good recovery rate of the observation group was higher compared to the control group, and the difference had statistical significance (P<0.05). The incidence of complications in the observation group was lower than that of the control group, with a statistically significant difference (P<0.05).

**Conclusion:**

In the treatment of cerebral hemorrhage, minimally invasive intracranial hematoma aspiration and drainage facilitates the recovery of patients, promotes the improvement of neurological function, and has a high safety profile and an ideal prognostic quality.

## INTRODUCTION

Cerebral hemorrhage has a high clinical incidence, mostly in the middle-aged and elderly population. At present, China’s aging process is accelerating, the incidence rate of cerebral hemorrhage is increasing, and the trend of younger patients is becoming increasingly obvious, which may be related to the life pressure, diet structure, and living habits.^1^,^2^ Cerebral hemorrhage refers to the bleeding caused by the non-traumatic rupture of blood vessels in the parenchyma, which is acute and progressing rapidly. The mortality rate in the acute stage is as high as 30% to 40%. The pathogenesis of cerebral hemorrhage is related to the aging of blood vessels, hypertension, diabetes, hyperlipidemia, etc. Cerebral hemorrhage suddenly happens often when people make excessive exertion or have emotional excitement, and the early mortality rate is high. Moreover, patients usually have cognitive disorders and limb dysfunction after treatment, which seriously affects their daily life.^3^-^5^ Currently, treatment for cerebral hemorrhage is based on hematoma removal, which can improve the state of brain tissue damage, but there is no specific clinical treatment. The choice of surgical or pharmacological treatment for patients with hypertensive cerebral hemorrhage is still controversial. Randomized controlled trials have failed to provide evidence that surgical treatment is significantly better than conservative treatment, which may be related to the inconsistency in the grasp of surgical indications and the choice of surgical methods; therefore, the choice of treatment deserves further investigation.^6^,^7^ In recent years, with the development of minimally invasive technology, minimally invasive intracranial hematoma aspiration and drainage has been applied in the treatment of cerebral hemorrhage with the advantages of simple operation, small trauma, and less complications. This study compared the effect of conservative treatment and minimally invasive intracranial hematoma aspiration and drainage on patients with cerebral hemorrhage and evaluated the prognosis of patients. The purpose of this study was to further study the effect of minimally invasive surgery in the clinical treatment of hypertensive cerebral hemorrhage and provide a guidance for clinical treatment.^8^,^9^ The report is as follows.

## METHODS

This study included 78 patients with cerebral hemorrhage who were treated in our hospital between June 2018 and December 2019. The patients were randomly numbered and divided into two groups by drawing lots, 39 each. There were 23 males and 16 females in the control group; they aged 54-71 years (average (62.07±3.86) years); the admission time was 1-11 h (average (5.69±0.83) h); the hematoma volume was 23-52 mL (average (35.25±7.03) mL). There were 25 males and 14 females in the control group; they aged 55-71 years (average (62.16±3.83) years); the admission time was 1-10 h (average (5.62±0.87) h); the hematoma volume was 24-51 mL (average (35.22±6.96) mL). The difference between the two groups in general data had little influence on the results; hence, the results were comparable. This study has been approved by the medical ethics committee of our hospital (No.004510321208, dated 21^st^ March 2018), and the patients have signed informed consent.

### Inclusive criteria:

1. Being diagnosed by magnetic resonance imaging (MRI), computed tomography (CT) examination, and clinical symptom observation;
2. Taking no immunopreparations and hormone drugs within one month before admission; -
3. The admission time was shorter than 12 h
4. Glasgow Coma Scale (GCS) score > seven points.

### Exclusive criteria:

1. Having cerebral hemorrhage caused by vascular malformation hemorrhage, ruptured cerebral aneurysm, and traumatic hematoma;
2. Having coagulation dysfunction;
3. Having malignant tumor;
4. Having severe hepatic and renal dysfunction;
5. Poor compliance with the treatment.

The control group was treated with the traditional internal medicine conservative treatment method, including reducing intracranial pressure by dehydration, adjusting blood pressure, preventing continuous bleeding, anti-infection by antibiotic, symptomatic drug support therapy, and preventing complications. The observation group was treated with minimally invasive intracranial hematoma aspiration and drainage. The specific operation was as follows. Intracranial CT examination was performed to determine the hematoma position of the patient. The puncture location was determined according to the specific situation of the patient. The skin around the puncture point was disinfected. The patient was given local anesthesia. The YL-1 intracranial hematoma puncture needle was used. The drainage tube was inserted through the needle to reach the hematoma cavity. The syringe was connected to aspirate the liquid part. The flushing fluid, i.e., normal saline and low molecular heparin, was pushed into the internal part at a dosage of 250 mL and 5,000 U respectively, 4 ~ 5 mL each time. After the discharge liquor became completely clear, hematoma liquefier, i.e., urokinase, low molecular heparin, and normal saline, was injected at a dosage of 10,000 ~ 20,000 U, 2500 U, and 2 ~ 4 mL respectively. The drainage tube was clamped for four hours and then opened. The aseptic dressing was used for binding. The drainage bag was changed once three or four days. The needle was retained for once week.

The two groups were given rehabilitation nursing after treatment. The first content was psychological nursing. Patients were very anxious and scared after hemiplegia; therefore, the nurses actively reassured and guided the patients, letting them know that as long as they adhered to the treatment and do a good job in rehabilitation, the chances of restoring body and speech functions were high. The second content was limb rehabilitation training. In order to avoid muscle atrophy and joint contracture, nursing staff actively assisted the patients in the early passive limb training, including hand, wrist, elbow, and shoulder, and lower limb training included toe, ankle, knee joint, and wrist joint. The healthy side was trained first, and then the affected side was trained. The training range was from small to large. The third content was daily life ability training. The nursing staff guided the patients to train daily living skills from simple to difficult, and to exercise their ability of coordinating hands and doing fine movements. For example, nurses guided the patients to do palm to palm and finger to finger, grip, button up, etc., and then gradually transit to dress clothes, wash face, wash clothes, etc.

### Observation indexes:

Efficacy evaluation criteria:^10^ Markedly effect: the patient had clear consciousness and could conduct daily affairs normally, and the limb muscle strength was higher than or equal to level III; effective: after treatment, the consciousness of the patient was slightly unclear, and the limb muscle strength evaluation was below level III; ineffective: the patient had unclear consciousness that has threatened life safety. Total effective rate = (number of markedly effective cases + number of effective cases)/total number of cases × 100%. The degree of neurological function defect was evaluated by National Institutes of Health Stroke Scale (NIHSS).^11^ The total score was 42 points. The higher the score was, the more the injured parts was, and the more severe the injury was. Prognosis quality assessment:^12^ The prognosis quality was evaluated six months after treatment. According to Glasgow Outcome Scale (GOS), patient who restored normal life and had good recovery was evaluated as five points, patient who had moderate disability but could live independently was evaluated as four points, patient who had severe disability and could not live by himself was evaluated as three points, patient who had sleep cycles and eye movements but kept a persistent vegetative state for a long time was evaluated as two points, and patient who died was evaluated as one point. The complication which occurred in two groups were recorded during the treatment, i.e., number of cases of intracranial infection, pulmonary infection, urinary tract infection, gastrointestinal bleeding, and cerebral hemorrhage enlargement (an increase in hematoma volume of 6 ml or 33% of the original volume).

### Statistical analysis:

SPSS 23.0 statistical software was used for data analysis. The measurement data were expressed as mean±SD; the non-independent sample t-test was used for comparison between groups, and paired sample t-test was used for comparison within groups. The count data were expressed as percentage (%), and the comparison was performed using Chi-square test. Difference was considered statistically significant if P<0.05.

## RESULTS

The total effective rate of clinical treatment in the observation group was significantly higher than that in the control group, and the difference was statistically significant (P<0.05, Table-I).The NIHSS scores of the two groups decreased after treatment. Moreover, the NIHSS score of the observation group was lower than that of the control group, and the difference was statistically significant (P<0.05, Table-II).The good recovery rate of the observation group was higher than that of the control group, and there was a statistically significant difference (P<0.05, Table-III).The incidence of complications in the observation group was lower than that of control group, and the difference was statistically significant (P<0.05, Table-IV).

**Table I:** Clinical efficacy between the two groups [n(%)].

Group	Observation group	Control group	X^2^	P
Markedly Effective	21(53.85)	13(33.33)	-	-
Effective	17(43.59)	15(38.46)	-	-
Ineffective	1(2.56)	11(28.21)	-	-
Total Efficiency	38(97.44)	28(71.89)	14.539	<0.05

**Table II:** NIHSS scores before and after treatment in the two groups (Mean±SD).

Group	Observation group	Control group	X^2^	P
Before Treatment	37.16±4.05	37.18±4.02	0.045	>0.05
After Treatment	12.44±1.66	17.27±1.95	12.139	<0.05
t	27.146	35.493	-	-
P	<0.05	<0.05	-	-

**Table III:** Good recovery rate between the two groups [n(%)].

Group	Observation group	Control group	X^2^	P
One Point	0(0.00)	2(5.13)	-	-
Two Points	3(7.69)	5(12.82)	-	-
Three Points	2(5.13)	4(10.26)	-	-
Four Points	3(7.69)	7(17.95)	-	-
Five Points	31(79.49)	21(53.85)	4.976	<0.05

**Table IV:** Complications between the two groups during treatment [n(%)].

Group	Observation group	Control group	X^2^	P
Intracranial infection	1(2.56)	0(0.00)	-	-
Urinary tract infection	0(0.00)	3(7.69)	-	-
Pulmonary infection	1(2.56)	3(7.69)	-	-
Gastrointestinal bleeding	1(2.56)	4(10.26)	-	-
Cerebral hemorrhage enlargement	0(0.00)	1(2.56)	-	-
Total incidence rate	3(7.69)	11(28.21)	5.564	<0.05

## DISCUSSION

Cerebral hemorrhage is one of the main diseases threatening the health of middle-aged and elderly people in China. It is caused by factors such as excessive exertion and emotional excitement. It develops rapidly and has a high early mortality rate, which is one of the main causes of death in elderly patients.^13^ At present, the development of medical operation level in China has made effective control of the mortality of cerebral hemorrhage. However, patients with cerebral hemorrhage are often accompanied by poor prognosis, and the amount of hemorrhage and hematoma directly affects the prognosis of patients. Therefore, the clinical treatment should pay attention to bleeding control and hematoma clearance work.

Traditional drug therapy mainly focuses on regulating blood pressure, lowering cranial pressure, and preventing infection, thus to achieve anti-inflammation, hemostasis, and blood stasis removal.^14^,^15^ However, for patients with cerebral hemorrhage, the risk of bleeding is very high and can lead to speech or mobility impairment in mild cases or coma or death in severe cases.^16^ In this study, the NIHSS score of the control group reduced to a small extent, the incidence of complications was 28.21%, and the good recovery rate was only 53.85%. Thus, it could be seen that traditional drug therapy is less effective in the recovery of patients with cerebral hemorrhage, in accordance with the results of previous studies.^17^,^18^

Minimally invasive intracranial hematoma aspiration and drainage is a minimally invasive surgical procedure that has been used in the treatment of cerebral hemorrhage in recent years. It has the advantages of being less invasive, fast-acting, and easy to perform. It can quickly remove the intracranial hematoma, reduce hematoma degradation products, and alleviate the damage to the lesion, thus to inhibit exacerbation, improve consciousness disorder, alleviate the occupying effect, and avoid the occurrence of cerebral hernia, which is more beneficial to the recovery of patients.^19^,^20^ Moreover, the application of urokinase can improve the biochemical enzyme activity to liquefy hematoma and make it being drained smoothly, further strengthening the protective effect on neurological function and avoiding secondary brain injury. In this study, the comparison of the short-term efficacy, safety, and quality of prognosis showed that the observation group was better than the control group, which fully demonstrated that minimally invasive intracranial hematoma aspiration and drainage had more effective treatment effect, was beneficial to the prognosis of patients, and could effectively avoid sequela. Ding et al. verified in a study that minimally invasive intracranial hematoma aspiration was more advantageous in eliminating hematoma and restoring neurological function and was conducive to improving the quality of life and reducing complications,^21^ which was similar to the results of this study.

Although the results of this study showed minimally invasive aspiration and drainage of intracranial hematoma was effective in the treatment of cerebral hemorrhage, this study still has the following shortcomings: there are long-term postoperative efficacy follow-ups, such as death cases, and randomized controlled studies with large sample sizes are needed at a later stage to further confirm the authenticity and accuracy of the results.

## CONCLUSION

Treatment of cerebral hemorrhage, minimally invasive intracranial hematoma aspiration facilitates the recovery of patients, can promote the recovery of neurological function, and has a high safety profile and an ideal prognostic quality. The therapy is worth recommendation.

### Authors’ Contribution:

**CD & YLJ:** Study design, data collection and analysis and are responsible for integrity of the study.

**YLJ & WS:** Manuscript preparation, drafting and revising.

**CD & JFB:** Review and final approval of manuscript.
